# Design of 2D Checkerboard Lattices B_12_H_8_ and B_10_C_2_H_6_ from Aromatic Icosahedral B_12_ and B_10_C_2_ Units

**DOI:** 10.3390/nano16171085

**Published:** 2026-08-31

**Authors:** Zhuo Sun, Shuai Wang, Hao-Ning Li, Jun-Hui Yuan, Hao Wang, Jiafu Wang

**Affiliations:** 1 China Ship Research and Development Academy, Beijing 100192, China; 2 School of Physics and Mechanics, Wuhan University of Technology, Wuhan 430070, China; 3 Wuhan Second Ship Design and Research Institute, Wuhan 430205, China

**Keywords:** Two-dimensional materials, B_12_ superatom, semiconductor, carrier mobility, ion migration, first-principles calculations

## Abstract

Assembling aromatic icosahedral superatoms into 2D lattices offers a promising route to function-oriented boron-based materials. Using first-principles calculations, we embed ${B_{12}H}_{12}^{2-}$ and its isoelectronic carbon-substituted analog B_10_C_2_H_12_ into a checkerboard framework, yielding two novel monolayers, *o*-B_12_H_8_ and *o*-B_10_C_2_H_6_. Both exhibit excellent kinetic, thermal, and mechanical stability, along with pronounced mechanical anisotropy and near-zero Poisson’s ratio. Electronic structure calculations reveal wide bandgaps of 4.75 and 4.63 eV, respectively. Remarkably, *o*-B_10_C_2_H_6_ achieves high carrier mobilities—up to 8440 cm^2^V^−1^s^−1^ for electrons and 8590 cm^2^V^−1^s^−1^ for holes—with strong anisotropy. Moreover, both materials show low migration barriers for alkali metal ions, highlighting their potential as ion conductors or electrode interfaces. This work establishes a novel design paradigm that constructs 2D checkerboard lattices from B_12_ and B_10_C_2_ superatom building blocks, laying a robust theoretical groundwork for subsequent investigations in this field.

## 1. Introduction

Two-dimensional (2D) materials, with their atomic-scale thickness and unique quantum confinement effects, have continuously led the research frontiers of condensed matter physics and materials science over the past decade [1]. Beginning with the first mechanical exfoliation of graphene, layered materials such as transition metal dichalcogenides, hexagonal boron nitride, and black phosphorus have successively entered the spotlight, exhibiting high carrier mobilities, excellent mechanical flexibility, and highly tunable electronic structures that are difficult to achieve simultaneously in conventional bulk materials [2,3,4,5]. More importantly, the symmetry breaking, weak interlayer van der Waals interactions, and fully exposed surfaces inherent to the two-dimensional limit endow these systems with distinctive advantages in fields such as logic devices, optoelectronics, energy conversion, and sensing [6,7,8,9]. At present, the core driving force in 2D materials research has progressively shifted from the passive exfoliation of naturally occurring layered crystals toward the on-demand design of structural units with targeted functionalities and the construction of entirely artificial 2D lattices, thereby motivating systematic exploration of novel building blocks and design strategies.

Against this backdrop, constructing 2D materials using superatoms as building blocks is emerging as a highly promising rational design pathway [10]. Superatoms are stable clusters that possess atom-like electronic configurations with closed electronic shells, uniform sizes, and predictable chemical behavior, enabling the collective electronic structure characteristics of the clusters to be transplanted into macroscopically ordered condensed-matter systems [11]. Among the reported studies on 2D superatom materials, fullerenes such as C_60_, C_24_ or C_20_, with their high symmetry and functionalizable carbon cage surfaces, are frequently employed as zero-dimensional superatom building blocks to construct 2D monolayers with novel electronic properties and superconducting phenomena through polymerization or metal doping [12,13,14,15]. On the other hand, B_12_ clusters based on icosahedral architecture, by virtue of their unique bonding characteristics arising from electron-deficient multicenter bonds and excellent thermodynamic stability, constitute another core class of building blocks for constructing boron-based and boron–carbon-based 2D frameworks [16,17,18]. At both experimental and theoretical levels, several 2D lattices based on fullerenes or boron clusters have been successfully predicted or synthesized, revealing a rich spectrum of physical phenomena ranging from Dirac electrons and topological states to high-specific-capacity electrode materials [19,20,21,22].

From the perspective of materials design, we aim to construct novel 2D systems with simple Bravais lattices. Currently, a variety of well-established lattice configurations are available for 2D periodic structures, including triangular, hexagonal (honeycomb), and checkerboard lattices. At the theoretical level, 2D materials based on B_12_ units in triangular and hexagonal lattices have already been explored [23,24]. However, the paradigm of systematically embedding boron-based superatom units into a checkerboard lattice framework remains entirely unexplored. How to achieve novel boron-based 2D materials that combine high stability with superior functional properties through isoelectronic substitution and geometric arrangement tuning remains an open and challenging issue.

To this end, this work combines the 2D checkerboard lattice framework with icosahedral boron-based superatoms ${B_{12}H}_{12}^{2-}$ and its isoelectronic carbon-substituted cluster B_10_C_2_H_12_, and through theoretical design constructs two novel 2D boron-based materials with distinctive structures—*o*-B_12_H_8_ and *o*-B_10_C_2_H_6_. Based on first-principles calculations, we first systematically investigate the lattice structures and phonon spectra of these two monolayers to confirm their energetic and kinetic stability. We then evaluate their Young’s moduli and Poisson’s ratios, revealing the mechanical anisotropy and rigidity characteristics of the systems. Regarding electronic properties, we perform detailed calculations of the band structures, density of states, and carrier mobilities of both materials. Furthermore, we explore the migration pathways and diffusion energy barriers of alkali metal ions within the planes of these two novel boron-based materials, preliminarily assessing their potential as ion conductors or electrode interface materials. This study aims to establish theoretical design principles from superatom building blocks to two-dimensional functional lattices, providing a solid physical foundation for subsequent experimental synthesis and application-oriented materials screening.

## 2. Methods

All calculations were performed using the VASP code [25,26], employing the projected-augmented-wave (PAW) method [27,28] within the generalized gradient approximation (GGA) [29]. The kinetic energy cutoff of plane wave basis was set to 500 eV. The exchange-correlation energy was expressed using the PBE functional [29]. The valence electron configurations were set as follows: 1*s*^1^ for H, 2*s*^2^2*p*^1^ for B, 2*s*^2^2*p*^2^ for C, 2*s*^1^ for Li, 3*s*^1^ for Na, 4*s*^1^3*p*^6^ for K. For structural optimization, a strict force criterion of 0.005 eV/Å in all directions were employed, in addition to a dense *k*-mesh (9 × 9 × 1). Furthermore, a vacuum slab of at least 15 Å was incorporated along the *z*-direction for all 2D monolayers to avoid interlayer interactions. Furthermore, the thermal stability of the 2D monolayers was evaluated through *ab initio* molecular dynamics (AIMD) simulations. These were carried out at various temperatures for 5 ps using a 3 × 3 × 1 supercell for 2D monolayers. Phonon dispersion calculations were performed using PHONOPY software [30]. To overcome the underestimation of bandgaps in semiconductors or insulators by the GGA-PBE method [31,32,33,34], we introduced the hybrid functional HSE06 [35] to evaluate the band gaps. Additionally, the intermolecular interactions were described using Grimme’s DFT-D3 dispersion correction [36]. The VASPKIT [37] and VESTA [38] software were employed in the process of data analysis.

## 3. Results

### 3.1. Structure and Stability

**Figure 1:**
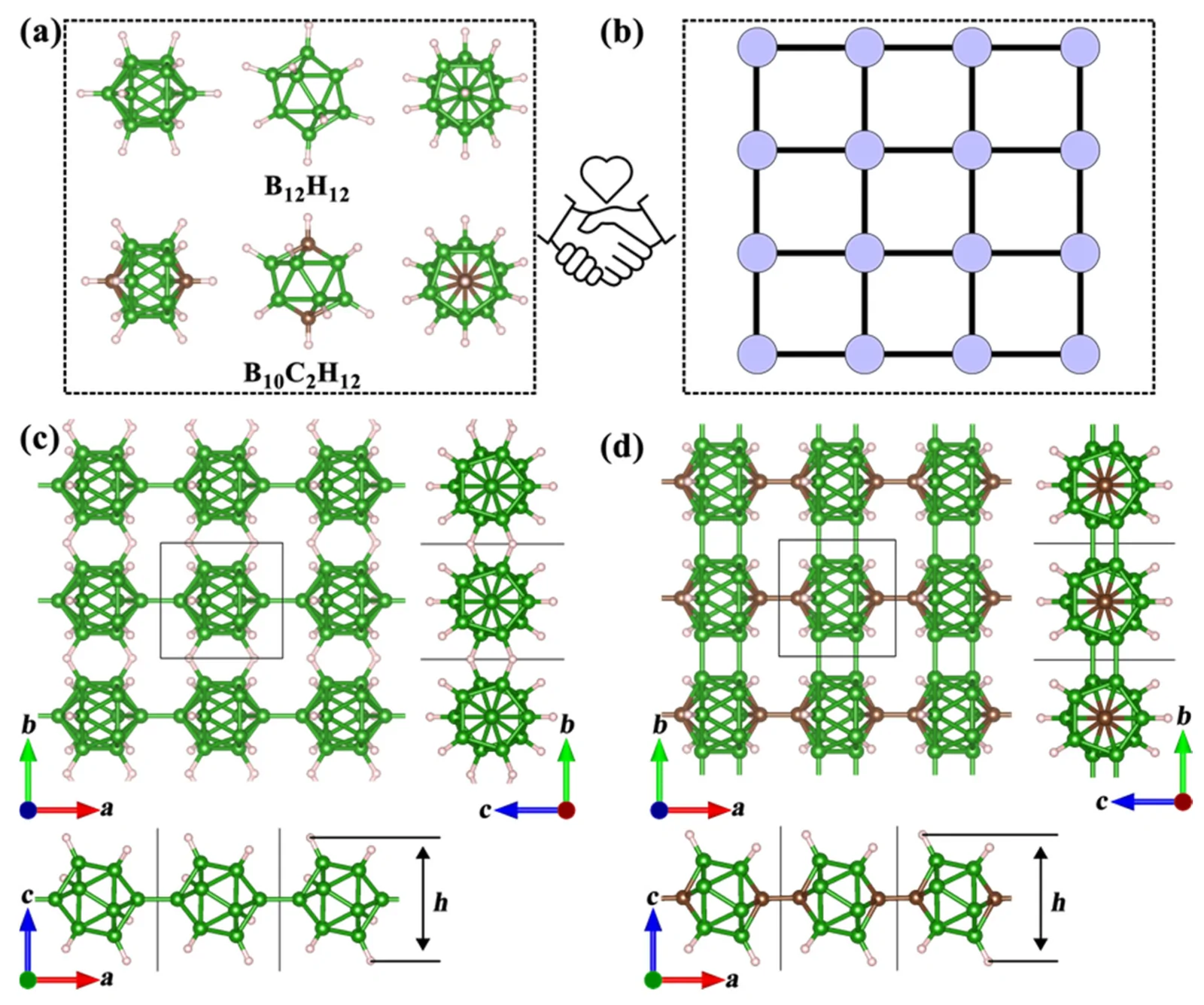
Design concepts of 2D *o*-B_12_H_8_ and *o*-B_10_C_2_H_6_. (**a**) Atomic structure of B_12_H_12_ and B_10_C_2_H_12_. (**b**) Schematic diagram of the 2D checkerboard lattice. Top and side view of 2D (**c**) *o*-B_12_H_8_ and (**d**) *o*-B_10_C_2_H_6_. The *h* represents the thickness of the atomic layer.

Figure 1a illustrates the closo-borane ${B_{12}H}_{12}^{2-}$ and its isoelectronic and isostructural analog, the closo-carborane B_10_C_2_H_12_. These two cluster species can serve as superatom building blocks for constructing one-, two-, and three-dimensional materials, and such assemblies have already been realized experimentally. In this work, we adopt a classic linear 2D lattice—the checkerboard lattice—as the design template, as shown in Figure 1b. The lattice sites of a checkerboard lattice can be occupied either by individual atoms or by atomic clusters, such as the ${B_{12}H}_{12}^{2-}$ and B_10_C_2_H_12_ clusters shown in Figure 1a. However, arranging these two clusters in a checkerboard pattern likely requires dehydrogenation. Based on this design strategy, we take ${B_{12}H}_{12}^{2-}$ and B_10_C_2_H_12_ as the initial building units and, by incorporating the geometric characteristics of the checkerboard lattice, design two novel 2D orthorhombic boron-based materials, termed *o*-B_12_H_8_ and *o*-B_10_C_2_H_6_, whose structures are presented in Figure 1c,d. In constructing these two new structures, we applied the same dehydrogenation strategy to ensure overall charge balance. Specifically, in *o*-B_12_H_8_, the hydrogen atoms at both ends along the a-direction are removed to expose boron atoms, facilitating the formation of stronger B-B bonds; along the b-direction, the hydrogen atoms are retained as shared bridging atoms to connect the building units. Owing to the specific symmetry of the checkerboard lattice, once the *o*-B_12_H_8_ structure was fixed, the possible substitution pattern upon replacing B_12_ with B_10_C_2_ becomes uniquely determined: the C–C bond must bridge the superatomic units along the a direction. The remaining issue is the bonding connectivity along the b direction. Initially, we adopted *o*-B_10_C_2_H_8_ as the starting structure for optimization in order to allow as much randomness as possible in the hydrogen desorption process. However, during structural relaxation, we observed that the B–H bonds bridging the superatomic units along the b direction spontaneously break, leading to the formation of B–B bonds; concurrently, the two released hydrogen atoms combine to form an H_2_ molecule and leave, thus yielding the final *o*-B_10_C_2_H_6_ structure, as shown in Figure 1d. It should be emphasized that, although the checkerboard lattice serves as our design prototype, the resulting structures after filling with superatoms no longer retain a perfect checkerboard pattern; instead, they belong to a distorted checkerboard lattice.

Table 1 lists the lattice parameters of the two designed novel 2D boron-based or boron–carbon-based monolayers, including the lattice constants (*a*/*b*), atomic layer thickness (*h*), and bond length (*l*) statistics. As noted earlier, although *o*-B_12_H_8_ and *o*-B_10_C_2_H_6_ originate from the checkerboard lattice design concept, the final structures deviate from a perfect checkerboard lattice due to the arrangement of superatoms and bonding requirements. *o*-B_12_H_8_ possesses lattice constants of 5.124 Å and 4.917 Å (*a* ≠ *b*), with an atomic layer thickness of 5.245 Å; its B-B bond lengths range from 1.703 to 1.836 Å, and its B-H bond lengths range from 1.186 to 1.333 Å, as shown in Figure S1a. In comparison, *o*-B_10_C_2_H_6_ has lattice constants of 4.719 Å and 4.711 Å, and an atomic layer thickness of 5.127 Å, all of which are smaller than the corresponding values for *o*-B_12_H_8_. In *o*-B_10_C_2_H_6_, the B-B bond lengths fall within the range of 1.759–1.805 Å, the B-C bond lengths lie between 1.703 and 1.755 Å, and the C-C bond and B-H bond lengths are 1.529 Å and 1.188/1.182 Å, as shown in Figure S1b. Overall, the basic building units in both monolayers largely retain their superatom structural characteristics.

In addition, we note that the corresponding bond lengths in *o*-B_12_H_8_ and *o*-B_10_C_2_H_6_ undergo significant changes, which can be mainly attributed to the introduction of C atoms (see Figure S1 for details). To this end, we combined electron localization function (ELF) and Bader charge analysis to explain the main differences between the two materials. As shown in Figure S1, compared with *o*-B_12_H_8_, the introduction of C atoms into *o*-B_10_C_2_H_6_ leads to a contraction of the C-B bonds relative to the original B–B bonds; meanwhile, the C-C bond length between superatoms along the a direction (1.529 Å) is also significantly shorter than the B-B bond (1.703 Å). This can be mainly attributed to the stronger C-C and B-C bonds compared with the B-B bonds. The ELF results in Figure S2 show that, overall, *o*-B_12_H_8_ and *o*-B_10_C_2_H_6_ exhibit similar charge localization characteristics. Along the a direction, both the B–B bonds in *o*-B_12_H_8_ and the C–C bonds in *o*-B_10_C_2_H_6_ between superatoms show strong covalent bonding features (with electrons localized at the bond center). However, along the b direction, the charge distribution differs markedly: in *o*-B_12_H_8_, electrons are mainly localized on H atoms, displaying ionic bonding characteristics; in contrast, in *o*-B_10_C_2_H_6_, electrons are localized at the B–B bond centers, showing typical covalent bonding. It is precisely the stronger covalent bonds along the axial direction in *o*-B_10_C_2_H_6_ that result in its higher Young’s modulus compared with *o*-B_12_H_8_ (see Section 3.2 for details). Figure S3 presents the Bader charge analysis results. Along the a direction, the amount of electron transfer between the B–B bonds in *o*-B_12_H_8_ is very small and far less than that of the corresponding C–C bonds in *o*-B_10_C_2_H_6_. The Bader charge distribution along the b direction is also consistent with the ELF analysis, further confirming that the B–B covalent bonds in *o*-B_10_C_2_H_6_ are stronger than the B–H bonds in *o*-B_12_H_8_. It is precisely these structural differences that lead to the differences in stability and mechanical properties between the two materials.

**Figure 2:**
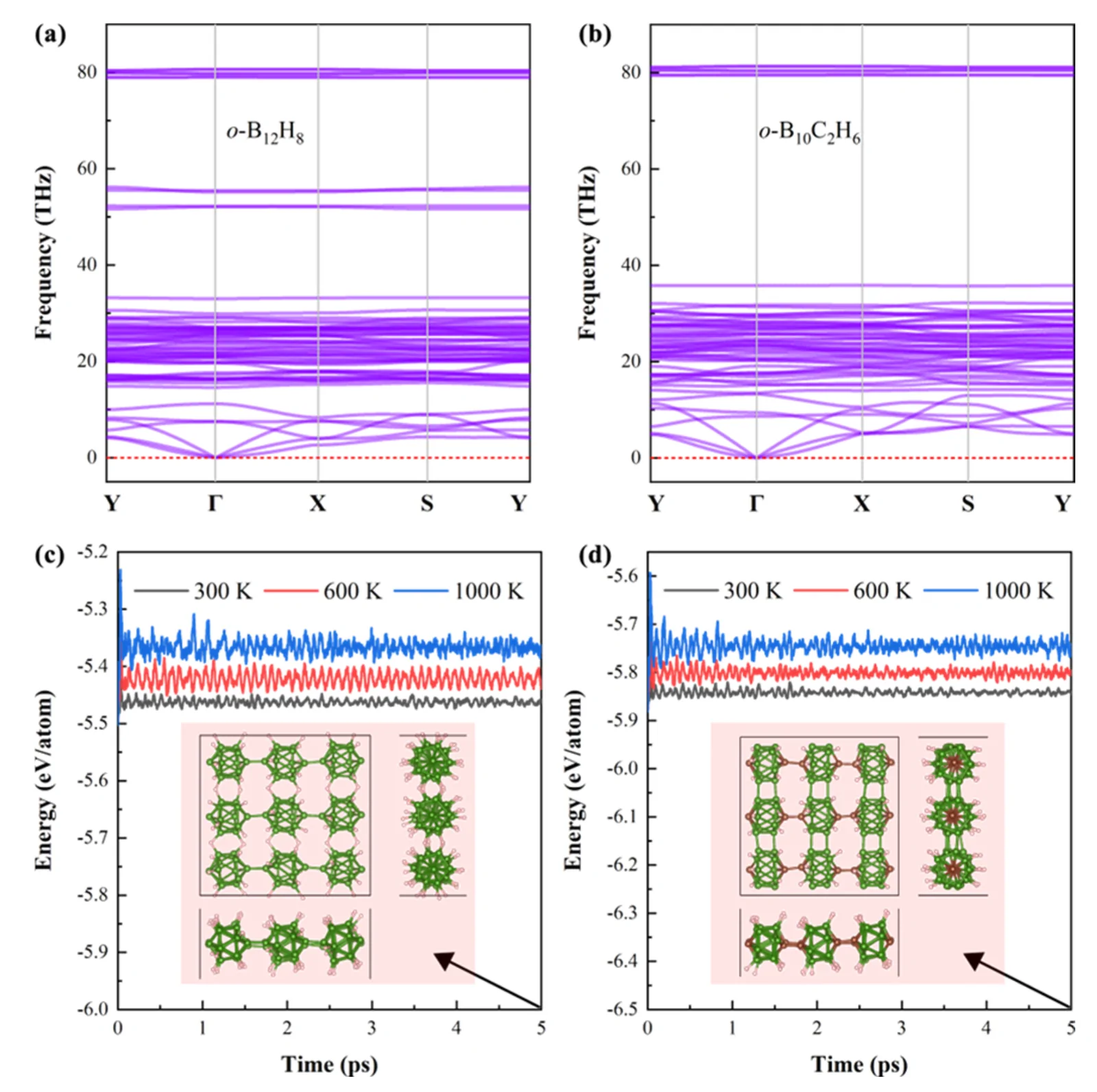
Phonon dispersions of (**a**) *o*-B_12_H_8_ and (**b**) *o*-B_10_C_2_H_6_. Results of AIMD simulations for (**c**) *o*-B_12_H_8_ and (**d**) *o*-B_10_C_2_H_6_ at different simulated temperatures. The insets show the final crystal structures of *o*-B_12_H_8_ and *o*-B_10_C_2_H_6_ at the end of the simulation time at 1000 K.

After performing high-precision structural optimization on the designed structures, we first evaluated their stability, which serves as the foundation for subsequent investigations. Figure 2 presents the phonon spectra of *o*-B_12_H_8_ and *o*-B_10_C_2_H_6_, together with their molecular dynamics simulation results at 300 K, 600 K, and 1000 K. As shown in Figure 2a,b, the phonon spectra of both structures are free of imaginary frequencies, indicating their excellent kinetic stability. During the 5 ps AIMD simulations, both systems exhibit good energy stability with only minor energy fluctuations. Although the total energy oscillations intensify with increasing simulation temperature, neither structure undergoes collapse by the end of the simulations; only partial breaking of B-B bonds in the B_12_ or B_10_C_2_ superatoms is observed, and the overall structural thermal stability is well maintained. It should be noted that the AIMD simulation time employed in this work is only 5 ps; therefore, the thermal stability at 1000 K still requires further verification through longer simulations (e.g., extending to 20 ps or more). Nevertheless, the current results are sufficient to confirm the thermal stability characteristics of *o*-B_12_H_8_ and *o*-B_10_C_2_H_6_ in the range from room temperature to moderate temperatures. Finally, we assessed their mechanical stability based on the Born-Huang criteria ($C_{11}C_{22}-C_{12}^{2}>0$, and $C_{66}>0$) [39]. The calculated independent elastic constants *C*_11_, *C*_22_, *C*_12_, and *C*_66_ of *o*-B_12_H_8_ are 118.92 N/m, 133.14 N/m, −1.15 N/m, and 20.87 N/m, respectively, while the corresponding values for *o*-B_10_C_2_H_6_ are 194.15 N/m, 233.69 N/m, 1.92 N/m, and 36.70 N/m. Clearly, the elastic constants of both structures satisfy the Born-Huang criteria, confirming their excellent mechanical stability. In addition, using β-B, graphite, and H_2_ as the reference phases, the formation energies of *o*-B_12_H_8_ and *o*-B_10_C_2_H_6_ are calculated to be −0.139 eV/atom and −0.004 eV/atom, respectively. The negative values indicate that both are exothermic relative to the elemental phases, i.e., thermodynamically favorable. However, the formation energy of *o*-B_10_C_2_H_6_ is very close to zero, implying that additional energy input may be required for its experimental stabilization. It should be noted that a rigorous determination of thermodynamic stability generally requires convex-hull analysis based on a complete phase diagram; such calculations have not been performed in this work, and the relevant conclusions therefore await further verification. The above results confirm that the two novel 2D boron-based and boron–carbon-based monolayers designed in this work can exist as free-standing layers, which undoubtedly lays a solid theoretical foundation for their potential experimental synthesis. Encouraged by the successful experimental synthesis of 2D fullerene structures [12,40], we believe that 2D materials based on boron icosahedral superatoms can also be successfully realized in the laboratory in the near future.

### 3.2. Young’s Modulus and Poisson’s Ratio

**Figure 3:**
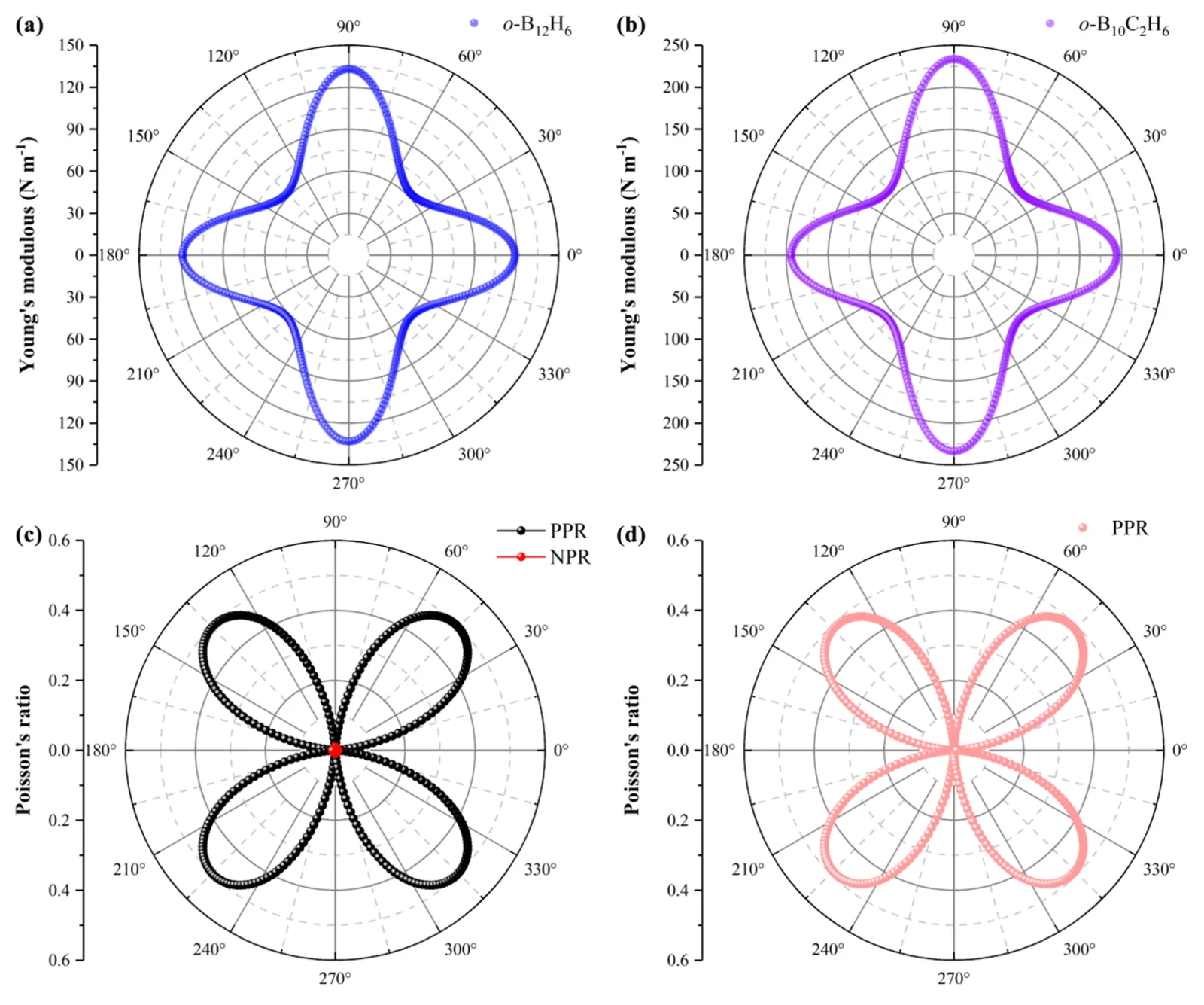
Calculated angle-dependent Young’s modulus of (**a**) *o*-B_12_H_8_ and (**b**) *o*-B_10_C_2_H_6_. Calculated angle-dependent Poisson’s ratio of (**c**) *o*-B_12_H_8_ and (**d**) *o*-B_10_C_2_H_6_.

Based on the independent elastic constants obtained from the mechanical stability assessment, we further calculated the angle-dependent Young’s modulus and Poisson’s ratio of *o*-B_12_H_8_ and *o*-B_10_C_2_H_6_; detailed calculation procedures are provided in Supplementary Material Note S1. As shown in Figure 3a,b, owing to their structural similarity, the Young’s moduli of *o*-B_12_H_8_ and *o*-B_10_C_2_H_6_ exhibit the same variation trend and both display anisotropic characteristics. The axial Young’s moduli of *o*-B_12_H_8_ are 118.91 N/m and 133.13 N/m, respectively, while its minimum value of 62.50 N/m occurs along the diagonal directions (44°/136°), amounting to only about half of the axial values. For *o*-B_10_C_2_H_6_, the axial Young’s moduli are 194.13 N/m and 233.67 N/m, and the minimum value of 109.18 N/m is likewise obtained along the diagonal directions (44°/136°), also approximately half of the axial values. Overall, under comparable conditions, the Young’s modulus of *o*-B_10_C_2_H_6_ is consistently larger than that of *o*-B_12_H_8_, indicating higher stiffness, which is attributed to the bonding differences revealed above: the C–C bonds along the a direction and the B–B bonds along the b direction in *o*-B_10_C_2_H_6_ are both stronger than the corresponding B–B and B–H bonds in *o*-B_12_H_8_. Compared with several common 2D materials, the Young’s moduli of *o*-B_12_H_8_ and *o*-B_10_C_2_H_6_ are lower than that of graphene (~340 N/m) [41], comparable to that of MoS_2_ (~200 N/m) [42], and markedly higher than that of black phosphorus (~40 N/m) [43], demonstrating that these two novel materials possess moderate Young’s moduli.

Subsequently, we calculated the Poisson’s ratios of the two materials to evaluate their response characteristics to stresses applied in different directions. As shown in Figure 3c,d, the Poisson’s ratio distribution trends of *o*-B_12_H_8_ and *o*-B_10_C_2_H_6_ are also highly similar. Notably, *o*-B_12_H_8_ exhibits a slight negative Poisson’s ratio (NPR) in the vicinity of the axial directions, with maximum negative values of only −0.009 and −0.01 along the a and b directions, respectively. The NPR of *o*-B_12_H_8_ is much smaller than that of *o*-B_12_N_2_ (−0.025/−0.045) [16], GaPS_4_ (−0.033) [44] and tetra-silicene (−0.044/−0.055) [45]. Such small negative Poisson’s ratios would be difficult to observe and exploit in practical applications and are therefore of limited practical significance; however, they indirectly reflect the near-zero Poisson’s ratio characteristic of *o*-B_12_H_8_ along the axial directions. Similarly, the Poisson’s ratios of *o*-B_10_C_2_H_6_ along the a and b axes are merely 0.008 and 0.01, respectively. A near-zero Poisson’s ratio implies that the material undergoes almost no transverse deformation under stress—that is, when stretched or compressed in one direction, the dimensions in the perpendicular direction remain essentially unchanged—a property of considerable value for certain specialized fields [46]. As the stress direction deviates from the axes, the Poisson’s ratios of *o*-B_12_H_8_ and *o*-B_10_C_2_H_6_ display a butterfly-shaped distribution and gradually increase, reaching their maximum values of 0.498 and 0.490, respectively, near the diagonal directions (46°/134°), remarkably close to 0.5. This indicates that in these directions, both materials approach an incompressible state, in which the volume remains nearly constant during deformation. In addition, compared with 2D borophenes or borides also constructed from B_12_ superatoms, such as *m*-B_24_/*o*-B_24_ [19], *h*-B_12_O_2_H_6_/B_12_S_2_H_6_ [17], and *h*-B_12_X_2_ (X = N, P, As) [16], the *o*-B_12_H_8_ and *o*-B_10_C_2_H_6_ in this work exhibit a moderate-to-high Young’s modulus but a larger Poisson’s ratio. This indicates that different lattice types and assembly modes can significantly affect the mechanical properties of the materials, and the detailed data are provided in Table S1 of the Supplementary Material. In summary, *o*-B_12_H_8_ and *o*-B_10_C_2_H_6_ possess moderate Young’s moduli, near-zero Poisson’s ratios along the axial directions, and Poisson’s ratios close to 0.5 near the diagonal directions, which undoubtedly lays a structural foundation for their applications in the field of mechanoelectronics.

### 3.3. Electronic Properties

**Figure 4:**
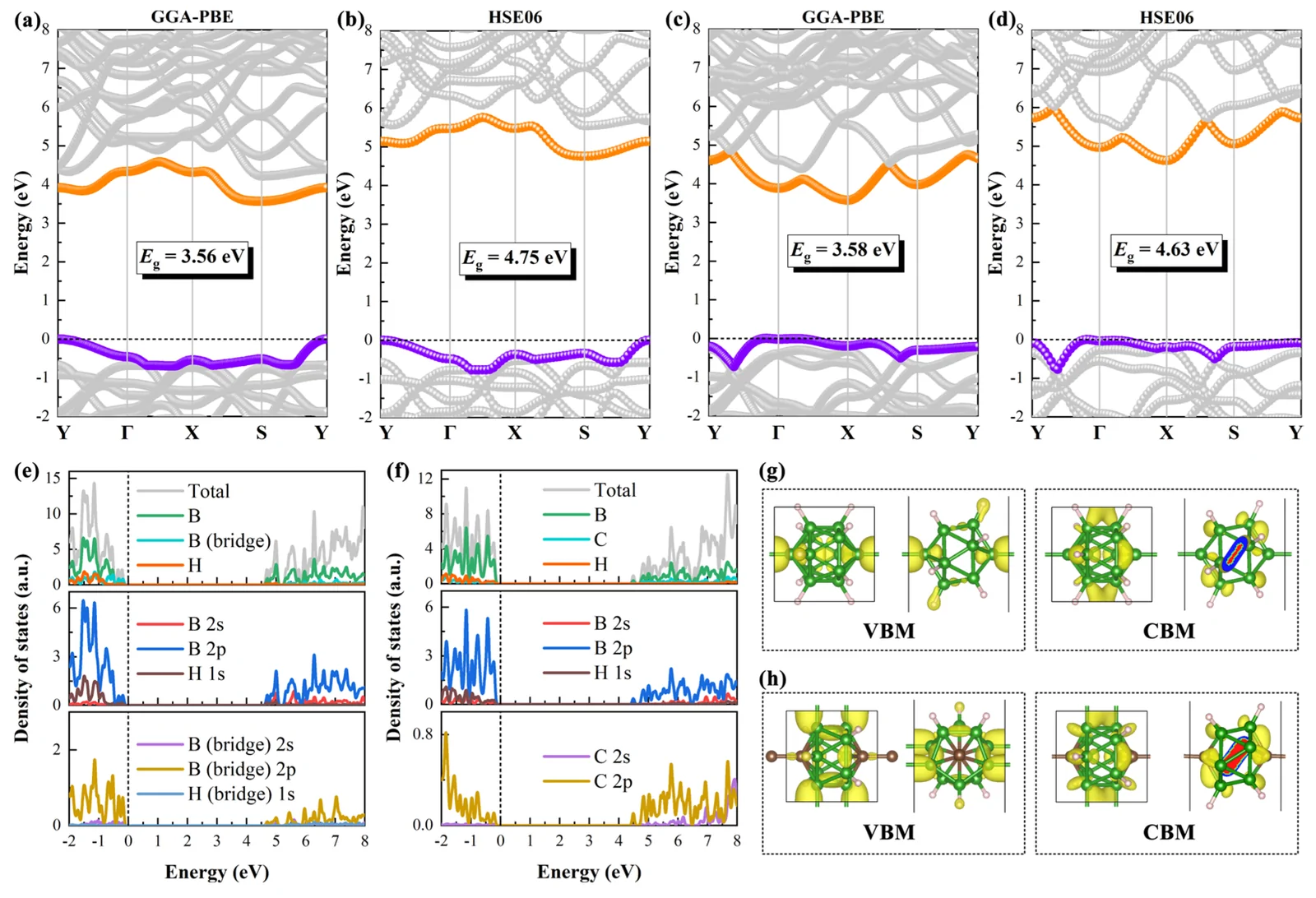
Electronic band structures of (**a**,**b**) *o*-B_12_H_8_ and (**c**,**d**) *o*-B_10_C_2_H_6_ calculated using the GGA-PBE and hybrid functional HSE06. Density of states of (**e**) *o*-B_12_H_8_ and (**f**) *o*-B_10_C_2_H_6_ based on HSE06 method. The corresponding partial charge density of VBM and CBM for (**g**) *o*-B_12_H_8_ and (**h**) *o*-B_10_C_2_H_6_.

We next focus on the electronic structures of *o*-B_12_H_8_ and *o*-B_10_C_2_H_6_, which are key to their potential application in high-performance electronic devices. Figure 4a–d present the band structures calculated at both the GGA-PBE and HSE06 levels. The results show that both *o*-B_12_H_8_ and *o*-B_10_C_2_H_6_ are wide-band-gap indirect semiconductors. The band gap of *o*-B_12_H_8_ is 3.56 eV (GGA-PBE) and 4.75 eV (HSE06), while that of *o*-B_10_C_2_H_6_ is 3.58 eV (GGA-PBE) and 4.63 eV (HSE06); the two materials thus exhibit comparable band gaps. It is noteworthy that the band gaps of *o*-B_12_H_8_ and *o*-B_10_C_2_H_6_ are significantly larger than those of *m*-/*o*-B_24_ (0.568–1.296 eV) [19], which are composed solely of B_12_ superatoms, and those of *h*-B_12_X_2_ (X = N, P, As) bridged by group V elements (0.995–1.128 eV) [16], but slightly smaller than that of *h*-B_12_(O/S)_2_H_6_ (4.927–5.257 eV) [17]. This indicates that the intrinsic structural features of the superatoms and their assembly modes can significantly affect the electronic structures of the predicted systems. More detailed data are provided in Table S1 of the Supplementary Material. For *o*-B_12_H_8_, the valence band maximum (VBM) lies at the Y point of the Brillouin zone, and the conduction band minimum (CBM) is along the S–X direction near S. For *o*-B_10_C_2_H_6_, the VBM is along the G–Y direction close to G, and the CBM is at the X point. The GGA-PBE and HSE06 band structures differ mainly in the magnitude of the band gap, while the band morphologies remain similar. Therefore, we adopt GGA-PBE for the subsequent calculation of carrier mobilities in both materials.

In Figure 4e,f, we present the calculated partial density of states (PDOS) contributions for *o*-B_12_H_8_ and *o*-B_10_C_2_H_6_, respectively. The results indicate that the valence band of *o*-B_12_H_8_ is predominantly contributed by the 2p orbitals of B atoms, with a notable contribution also arising from the 2p orbitals of the bridging B atoms (labeled as B bridge) that connect adjacent B_12_H_8_ building blocks. The conduction band is likewise dominated by B 2p orbitals, although the primary contribution in this case originates from the non-bridging B atoms. Additionally, the DOS reveals a strong sp orbital hybridization between B and H atoms. For *o*-B_10_C_2_H_6_, the valence band is overwhelmingly dominated by the 2p orbitals of B atoms within the B_10_C_2_H_6_ units, while the contribution from C atoms is substantially smaller; the conduction band exhibits the same orbital contribution trend.

Subsequently, we display the charge density distributions of the VBM and CBM for *o*-B_12_H_8_ and *o*-B_10_C_2_H_6_ in Figure 4g and Figure 4h, respectively. It is visually evident that the VBM of *o*-B_12_H_8_ primarily arises from the bridging B atoms, along with partial contributions from certain B and H atoms within the superatoms. In contrast, the CBM charge density is mainly concentrated in the inter-superatom regions along the b direction and on the non-bridging B atoms within the superatoms. For *o*-B_10_C_2_H_6_, the VBM charge density is mainly localized on the bridging B–B bonds along the b direction that connect adjacent superatoms, with a minor fraction distributed on the B and H atoms within the superatoms. The CBM charge distribution resembles that of *o*-B_12_H_8_, being likewise concentrated on the bridging B–B bonds along the b direction and on the other non-bridging B atoms within the superatoms.

We then calculated the carrier mobilities of *o*-B_12_H_8_ and *o*-B_10_C_2_H_6_ based on deformation potential theory. It should be noted that the Bardeen–Shockley deformation potential theory [47] accounts solely for phonon-limited carrier mobility, without incorporating other scattering mechanisms. Consequently, its predictions are idealized theoretical estimates, and the experimentally achievable mobilities may be significantly lower. Furthermore, the elastic constants, effective masses, and deformation potential constants used in the calculations are derived from fitting procedures, and the inherent fitting uncertainties can propagate into the final results. Hence, these data should be interpreted with due caution. In addition, the standard Bardeen–Shockley approach is formulated for homogeneous isotropic media and tends to overestimate mobilities in strongly anisotropic systems. Therefore, in this work, we adopted the modified Bardeen–Shockley method proposed by Lang et al. [48] to evaluate the carrier mobilities of *o*-B_12_H_8_ and *o*-B_10_C_2_H_6_. Details of the methodology are provided in Supplementary Material Note S2, and this approach has been employed in several previous studies [49,50,51,52].

Table 2 summarizes the effective masses (m^*^) of electrons and holes along different transport directions obtained from deformation potential theory, the absolute deformation potential constants (|*E*_1_|) derived from band-edge fitting, and the 2D elastic constants (*C*_2D_) extracted from strain–energy fitting (see Figure S4). The results show that the electron effective mass of *o*-B_12_H_8_ is significantly larger than the hole effective mass, which is attributable to its relatively flat conduction band. The fitted deformation potential constants are generally large, except for the hole along the b direction, with the electron deformation potential constant along b reaching as high as 13.698 eV. The combination of large carrier effective masses and large deformation potential constants leads to very low electron mobilities for *o*-B_12_H_8_, ranging from 2.806 to 7.487 cm^2^ V^−1^ s^−1^. Such low electron mobility would severely limit its potential application in high-performance electronic devices. In contrast, its hole mobility, ranging from 249.583 to 322.265 cm^2^ V^−1^ s^−1^, is slightly higher than that of MoS_2_ (~200 cm^2^V^−1^s^−1^) [53], indicating promising application potential.

Compared with *o*-B_12_H_8_, the carrier effective masses of *o*-B_10_C_2_H_6_—except for the hole along the a direction—are substantially smaller, falling in the range of 0.402–0.793 *m*_0_. Its deformation potential constants are also modest, with only the electron deformation potential constant along a being relatively large (3.042 eV); all other deformation potential constants for both electrons and holes are below 1.098 eV. Combined with the large 2D in-plane elastic constants of *o*-B_10_C_2_H_6_, which are attributed to its strong covalent bonding along the axial directions, the electron and hole mobilities along the a direction reach 2111.652 and 819.208 cm^2^ V^−1^ s^−1^, respectively, while those along the b direction are even higher, at 8440.619 and 8590.306 cm^2^V^−1^s^−1^. These values far exceed that of MoS_2_ (~200 cm^2^V^−1^s^−1^) [53] and are comparable to those of black phosphorus (~10^4^ cm^2^V^−1^s^−1^) [54]. In addition, compared with other B_12_-based 2D materials listed in Table S1, such as *m*-/*o*-B_24_, *h*-B_12_X_2_ (X = N, P, As), and *h*-B_12_(O/S)_2_H_6_, *o*-B_10_C_2_H_6_ exhibits a higher electron mobility, whereas the electron mobility of *o*-B_12_H_8_ is relatively lower. Overall, although the wide band gaps of *o*-B_12_H_8_ and *o*-B_10_C_2_H_6_ may preclude their use in low-power logic devices, their high thermal stability and considerable carrier mobilities render them potentially suitable for applications in high-voltage, high-frequency, and high-temperature electronics.

### 3.4. Migration Behavior of Alkali Metal Ions

**Figure 5:**
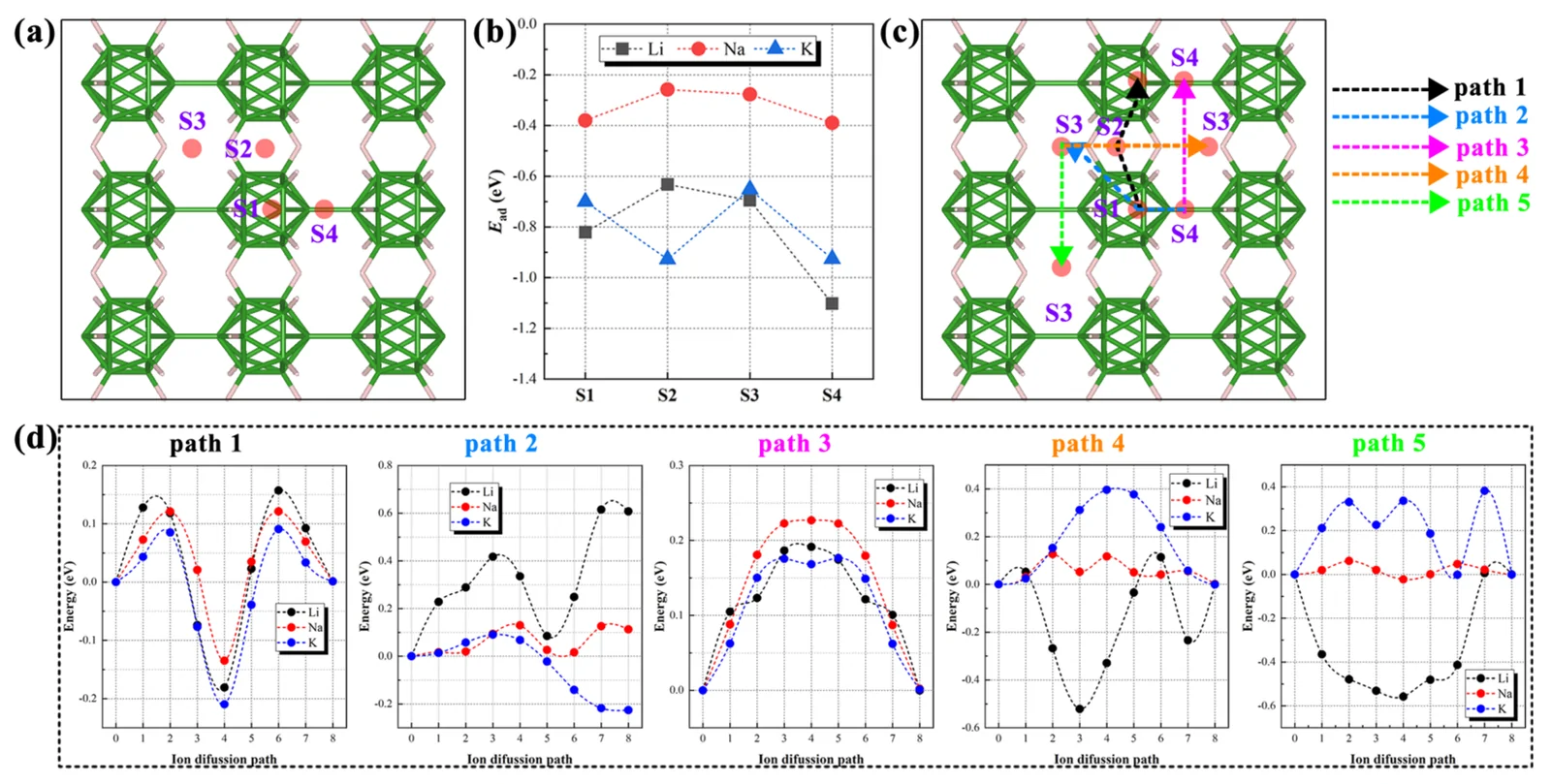
(**a**) Schematic illustration of the four inequivalent initial adsorption sites (S1–S4) on the *o*-B_12_H_8_ surface; (**b**) comparison of the optimized adsorption energies of Li^+^, Na^+^, and K^+^ at the four sites, respectively; (**c**) five representative ion migration paths designed based on the adsorption site configurations; (**d**) the corresponding energy barrier profiles for the five migration paths calculated using the CI-NEB method.

In the final part of this work, given that borophene and its derivatives generally exhibit good ionic conductivity, we further evaluated the migration characteristics of alkali metal ions on the surfaces of *o*-B_12_H_8_ and *o*-B_10_C_2_H_6_. First, for the *o*-B_12_H_8_ system, we identified four inequivalent initial adsorption sites on the surface of its 2 × 2 × 1 supercell based on structural symmetry, designated as S1, S2, S3, and S4 (Figure 5a). Subsequently, we placed Li^+^, Na^+^, and K^+^ at each site, performed structural relaxation, and calculated the corresponding adsorption energies (Figure 5b). The results show that all adsorption energies are negative (−1.1 to −0.2 eV/atom), indicating that the adsorption process is exothermic and can proceed spontaneously, with moderate adsorption energies. In addition, the adsorption energies at some sites are very close to each other. By examining the optimized atomic structures (Figure S5), we found that this is due to the significant site convergence of alkali metal ions after relaxation: for example, K^+^ initially placed at S1 and S4 converges to the same stable site, while that initially placed at S2 and S3 converges to another equivalent stable site, suggesting that the system possesses higher structural symmetry and thermodynamic stability.

Based on the above adsorption site calculations, we designed five migration paths for alkali metal ions on the *o*-B_12_H_8_ surface (Figure 5c). Path 1 is S1→S2→S1 (the ion starts from S1, passes through S2, and returns to the symmetric counterpart of S1); Path 2 is S4→S1→S3 (from S4 via S1 to S3); Path 3 is S4→S4 (migration to an adjacent S4 site via a lattice vacancy); Path 4 is S3→S3 (migration to an adjacent S3 site along the x direction); and Path 5 is also S3→S3 (migration to an adjacent S3 site along the y direction). We then calculated the migration barriers for these five paths using the CI-NEB method [55], with the results shown in Figure 5d and the corresponding CI-NEB migration images presented in Figure S6. Along Path 1, the migration barriers for Li^+^, Na^+^, and K^+^ are 0.338, 0.256, and 0.300 eV, respectively, which are generally low. Along Path 2, the barriers vary significantly among the three ions: Na^+^ exhibits the lowest barrier (0.130 eV), Li^+^ shows the highest (0.615 eV), and K^+^ falls in between (0.316 eV). Along Path 3, the barriers for the three ions are relatively close, at 0.191 eV (Li^+^), 0.227 eV (Na^+^), and 0.176 eV (K^+^). Along Path 4, the barrier for Li^+^ is 0.521 eV, that for Na^+^ is only 0.127 eV, and that for K^+^ is 0.396 eV. Along Path 5, Li^+^ has the highest barrier (0.558 eV), Na^+^ has the lowest (0.084 eV), and K^+^ has a barrier of 0.337 eV. Considering all five paths collectively, *o*-B_12_H_8_ is most favorable for Na^+^ migration (with the lowest barrier of only 0.084 eV), while Li^+^ migration is relatively difficult, and K^+^ lies in between. Therefore, based solely on the migration barriers of alkali metal ions on the *o*-B_12_H_8_ surface, this material exhibits promising potential for ionic conduction.

**Figure 6:**
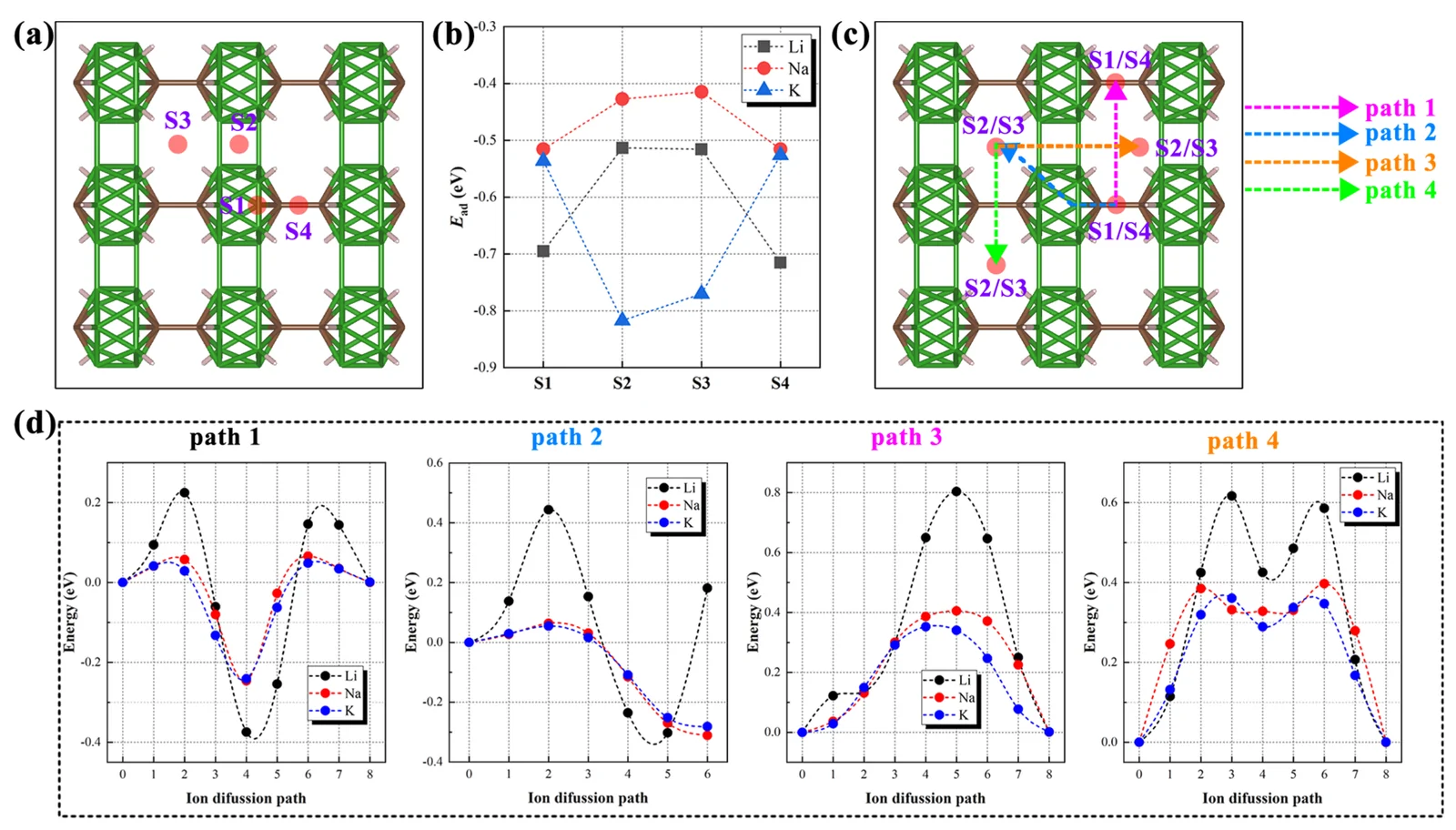
(**a**) Schematic illustration of the four inequivalent initial adsorption sites (S1–S4) on the *o*-B_10_C_2_H_6_ surface; (**b**) comparison of the optimized adsorption energies of Li^+^, Na^+^, and K^+^ at the four sites, respectively; (**c**) four representative ion migration paths designed based on the adsorption site configurations; (**d**) the corresponding energy barrier profiles for the four migration paths calculated using the CI-NEB method.

Subsequently, we investigated the adsorption and migration behaviors of alkali metal ions on the *o*-B_10_C_2_H_6_ surface using the same methodology. As shown in Figure 6a, *o*-B_10_C_2_H_6_ also possesses four adsorption sites (S1–S4), similar to *o*-B_12_H_8_. The optimized adsorption energies (Figure 6b) indicate that the adsorption of alkali metal ions on this surface is also exothermic (−0.82 to −0.41 eV), with the adsorption energies at S1 and S4, as well as those at S2 and S3, being close to each other. From the optimized atomic structures (Figure S7), it is evident that ions initially adsorbed at S1 and S4 spontaneously converge to the same equivalent site after relaxation, and the same occurs for those at S2 and S3. Consequently, the subsequent migration path planning is relatively simplified. Based on the above results, we designed four migration paths on the *o*-B_10_C_2_H_6_ surface (Figure 6c): Path 1 is S4→S4 (migration to an adjacent S4 site along the y direction); Path 2 is S4→S3; Path 3 is S3→S3 (migration to an adjacent S3 site along the x direction); and Path 4 is S3→S3 (migration to an adjacent S3 site along the y direction).

We then calculated the migration barriers for these four paths using the CI-NEB method (Figure 6d), with the final ion migration pathway diagrams presented in Figure S8. The results show that along Path 1, the barriers for Li^+^, Na^+^, and K^+^ are 0.599, 0.312, and 0.289 eV, respectively, exhibiting a decreasing trend. Along Path 2, Li^+^ shows the highest barrier (0.746 eV), while Na^+^ and K^+^ have comparable barriers (0.375 vs. 0.336 eV). The overall trend along Path 3 is similar to that of Path 2, with Li^+^ being the highest (0.804 eV), K^+^ the lowest (0.353 eV), and Na^+^ in between (0.405 eV). Along Path 4, Li^+^ still has the highest barrier (0.617 eV), followed by Na^+^ (0.397 eV), and K^+^ the lowest (0.361 eV). In summary, unlike *o*-B_12_H_8_, *o*-B_10_C_2_H_6_ exhibits the lowest migration barriers for K^+^ (0.289–0.361 eV) while showing relatively high barriers for Li^+^ (>0.6 eV), indicating its favorable K^+^ conductivity over the other two ions. In addition, we compared the migration barriers of alkali metal ions on the surfaces of *o*-B_12_H_8_ and *o*-B_10_C_2_H_6_ with the corresponding results for *m*-/*o*-B_24_ [19] and *h*-B_12_X_2_H_6_ (X = O, S) [17] from our previous work (see Table S1). The results show that the migration barriers of the two materials in this work are close to those of *m*-/*o*-B_24_ (0.16–0.89 eV/0.13–0.43 eV), but slightly higher than those of *h*-B_12_X_2_H_6_ (X = O, S) (0.15–0.45 eV/0.033–0.23 eV). In conclusion, both *o*-B_12_H_8_ and *o*-B_10_C_2_H_6_ surfaces exhibit moderate adsorption energies and favorable surface migration barriers for alkali metal ions, making them promising candidate materials as potential conductors for relevant ions.

Finally, we briefly note the limitations of this work. Although the precursors ${B_{12}H}_{12}^{2-}$ and B_10_C_2_H_12_ used in our theoretical predictions have been widely employed in experimental cluster assembly, suggesting that the two-dimensional structures designed here based on such superatoms possess a certain degree of experimental feasibility, it should be noted that direct synthetic routes toward this specific checkerboard topology remain to be explored, and the related discussion currently falls within the realm of theoretical prediction. We sincerely hope that future researchers in synthetic chemistry will be able to realize the design concepts proposed in this work experimentally.

## 4. Conclusions

In summary, we have theoretically designed two novel 2D boron-based monolayers—*o*-B_12_H_8_ and *o*-B_10_C_2_H_6_—by embedding icosahedral superatoms (${B_{12}H}_{12}^{2-}$^−^ and its isoelectronic B_10_C_2_H_12_ analog) into a checkerboard lattice. Comprehensive stability assessments confirm that both sheets are kinetically, thermally, and mechanically robust. They exhibit pronounced mechanical anisotropy and near-zero Poisson’s ratio along the axial directions, distinct from conventional 2D materials. Electronic structure calculations show that both are wide-bandgap semiconductors with bandgaps of 4.75 eV and 4.63 eV, respectively. Remarkably, *o*-B_10_C_2_H_6_ displays exceptionally high and anisotropic carrier mobilities, reaching up to 8440 cm^2^ V^−1^ s^−1^ for electrons and 8590 cm^2^ V^−1^ s^−1^ for holes. Additionally, low migration barriers for Li^+^, Na^+^, and K^+^ ions suggest that these materials are promising for ion conductors or electrode interfaces, with *o*-B_12_H_8_ favoring Na^+^ and *o*-B_10_C_2_H_6_ favoring K^+^ transport. This work demonstrates the feasibility of tuning band structures and ion channels via checkerboard topology, while the B_12_→B_10_C_2_ substitution shows that hetero-element incorporation can effectively modulate bonding, charge distribution, and band-edge orbital composition to optimize bandgap and mobility. Thus, this study goes beyond the prediction of two new materials—it validates the design strategies and offers a methodological framework for designing similar materials.

## Figures and Tables

**Table 1:** Calculated lattice constants *a*/*b* (Å), bond lengths *l* (Å), atomic layer thickness *h* (Å), and band gaps (in eV at the GGA-PBE and HSE06 levels) of *o*-B_12_H_8_ and *o*-B_10_C_2_H_6_.

Materials	*a*	*b*	*h*	*l*_B-B_	*l*_C-C_	*l*_B-C_	*l*_B-H_	$E_{g}^{PBE}$	$E_{g}^{HSE}$
*o*-B_12_H_8_	5.214	4.917	5.245	1.703~1.836	--	--	1.186~1.333	3.56	4.75
*o*-B_10_C_2_H_6_	4.719	4.711	5.127	1.759~1.805	1.529	1.703~1.755	1.182/1.188	3.58	4.63

**Table 2:** Calculated effective masses m^*^ (*m*_0_) of electrons (*e*) and holes (*h*), in-plane elastic modulus *C*_2D_ (N m^−1^), deformation potential constants |*E*_1_| (eV), and corresponding carrier mobilities μ (cm^2^V^−1^s^−1^) for *o*-B_12_H_8_ and *o*-B_10_C_2_H_6_. The carrier mobility results are calculated based on the methods proposed by Lang (left, underlined) and Bardeen-Shockley (right).

Materials	Carrier Type	$m_{a}^{*}$	$m_{b}^{*}$	|*E*_1*a*_|	|*E*_1*b*_|	$C_{a}^{2D}$	$C_{b}^{2D}$	$\mu_{a}^{2D}$	$\mu_{b}^{2D}$
*o*-B_12_H_8_	*e*	2.387	3.487	2.365	13.698	118.364	134.454	7.487/65.447	2.806/1.517
	*h*	0.660	1.081	6.271	0.582	118.364	134.454	249.583/114.988	322.265/9258.761
*o*-B_10_C_2_H_6_	*e*	0.793	0.402	3.042	0.452	193.032	233.044	2111.652/992.256	8440.619/107,033.810
	*h*	4.137	0.528	1.098	0.662	193.032	233.044	819.208/557.718	8590.306/14,513.224

## Data Availability

All data needed to evaluate the conclusions in the paper are present in the paper.

## Supplementary Materials

The following supporting information can be downloaded at: https://www.mdpi.com/article/10.3390/nano16171085/s1, Note S1: Formulas for Young’s modulus Y and Poisson’s ratio ν; Note S2: Details for carrier mobility calculation; Figure S1: The bond length of *o*-B_12_H_8_ and *o*-B_10_C_2_H_6_; Figure S2: The electron localization function of *o*-B_12_H_8_ and *o*-B_10_C_2_H_6_; Figure S3: The Bader charge analysis results of *o*-B_12_H_8_ and *o*-B_10_C_2_H_6_; Table S1: Calculated axis Young’s modulus (*Y*, N/m), axis Poisson’s ratio (*v*), band gaps (GGA-PBE/HSE06, eV), carrier mobilities (*m_x_*/*m_y_*, cm^2^V^−1^s^−1^) and Li/Na/K migration barriers (*E_ion_*, eV) of *o*-B_12_H_8_ and o-B_10_C_2_H_6_ MLs. The relevant parameters of the B_12_-based 2D materials proposed in our previous work are also listed in the table for comparison. Figure S4: The fitting curves of the in-plane elastic modulus *C*_2D_ and the deformation potential constant *E*_1_; Figure S5: Optimized configurations of Li^+^, Na^+^, and K^+^ at the various adsorption sites (S1–S4) on *o*-B_12_H_8_; Figure S6: Ion migration diagrams of Li^+^, Na^+^, and K^+^ along four preset migration paths on the *o*-B_12_H_8_ surface; Figure S7: Optimized configurations of Li^+^, Na^+^, and K^+^ at the various adsorption sites (S1–S4) on *o*-B_10_C_2_H_6_; Figure S8: Ion migration diagrams of Li^+^, Na^+^, and K^+^ along four preset migration paths on the *o*-B_10_C_2_H_6_ surface.
