# Supplementary material for: Design of 2D Checkerboard Lattices B12H8 and B10C2H6 from Aromatic Icosahedral B12 and B10C2 Units
Source: Nanomaterials (Basel). 2026 Aug 31;16(17):1085. doi: 10.3390/nano16171085 (PMC13567038; doi:10.3390/nano16171085)
Supplement: Supplementary file 1 [file nanomaterials-16-01085-s001.zip › nanomaterials-4502536-supplementary.pdf]

Supporting Information for

Design of 2D Checkerboard Lattices $B_{12}H_8$ and $B_{10}C_2H_6$ from Aromatic Icosahedral B_{12} and $B_{10}C_2$ Units

Zhuo Sun,[1,] Shuai Wang,[1] Hao-Ning Li,[2] Jun-Hui Yuan,[2,*] Hao Wang,[3,*] and Jiafu Wang,[2]

[1]China Ship Research and Development Academy, Beijing, 100192, China

[2]School of Physics and Mechanics, Wuhan University of Technology, Wuhan 430070, China

[3]Wuhan Second Ship Design and Research Institute, Wuhan 430205, China

[*]Corresponding Author

E-mail: yuanjh90@163.com (J.-H. Yuan); whuwanghao@163.com (H. Wang)

Note S1. Formulas for Young's modulus *Y* and Poisson's ratio *v*.

For the angle dependent in-plane Young's modulus (*Y*) and Poisson's ratio (υ) are calculated using the independent elastic constants (C_{11}, C_{22}, C_{12} and C_{66}). The calculation formula is as follows [1]:

$$Y(\theta) = \frac{C_{11}C_{22} - C_{12}^2}{C_{11}sin^4\theta + C_{22}cos^4\theta + \left(\frac{C_{11}C_{22} - C_{12}^2}{C_{66}} - 2C_{12}\right)cos^2\theta sin^2\theta}$$

$$\nu(\theta) = \frac{C_{12}(sin^4\theta + cos^4\theta) - (C_{11} + C_{22} - \frac{C_{11}C_{22} - C_{12}^2}{C_{66}})cos^2\theta sin^2\theta}{C_{11}sin^4\theta + C_{22}cos^4\theta + \left(\frac{C_{11}C_{22} - C_{12}^2}{C_{66}} - 2C_{12}\right)cos^2\theta sin^2\theta}$$

Note S2. Details for carrier mobility calculation.

The acoustic-phonon limited mobility can be obtained initially based on the deformation potential theory proposed by Bardeen and Shockley (BS for short)[2]. That is, if treating the semiconductor as a homogeneous media, the carrier mobility of 2D materials can be calculated by the following equation:

$$\mu_{\mathrm{2D}} = \frac{e\hbar^3 C_{\mathrm{2D}}}{k_{\mathrm{B}} T m^* m_{\mathrm{d}} (E_{\mathrm{l}}^{i})^2},$$

where $\hbar$ is the reduced Planck constant, k_{B} is the Boltzmann constant, m^* is the effective mass in the transport direction, m_{d} is the average effective mass determined by $m_{\mathrm{d}}=(m_a^* m_b^*)^{1/2}$, and *T* is the temperature (we use T = 300 K). The elastic modulus C_{2D} of the longitudinal strain in the propagation direction is derived from $(E - E_0)/S_0 = C_{\mathrm{2D}}(\Delta l / l_0)^2 / 2$, where *E* is the total energy of the 2D structure, and S_0 is the lattice area of the equilibrium supercell. The deformation potential constant E_{l}^{i} is defined as $E_{\mathrm{l}}^{i}=\Delta E_i / (\Delta l / l_0)$. Here ΔE_i is the energy change of the ith band under proper cell compression and dilatation (calculated using a step of 0.5%), l_0 is the lattice constant in the transport direction and Δl is the deformation of l_0. Such formula

may be used when the elastic constants and the deformation potential constants are relatively homogenous over the *a*-*b* plane for 2D semiconductors.

Nevertheless, as the deformation potential constants show great anisotropy, the above ultra-high mobilities are questionable. For instance, an electron experiences less scattering from the acoustic phonons from the *b*-direction, but the phonons from *a*-direction may impose strong scattering on the same electron. In a recent work by Lang *et al.*[3], a new formula for the acoustic phonon-limited mobility in 2D semiconductors was given, taking into the account of the anisotropic elastic constants or deformation potential constants:

$$\mu_{\mathrm{a}} = \frac{e\hbar^3}{k_{\mathrm{B}} T (m_a^*)^{3/2} (m_b^*)^{1/2}} \left(\frac{A + B - \sqrt{A^2 - B^2}}{B\sqrt{A^2 - B^2}} \right) \left(\frac{I + J - \sqrt{I^2 - J^2}}{J\sqrt{I^2 - J^2}} \right),$$

where the parameters A, B, I, J are defined as

$$A = \overline{E}_l^2 + \frac{\Delta E_l^2}{2}, \quad B = E_l \Delta E_l$$

$$I = \frac{1}{\sqrt{\overline{C}^2 - \Delta C^2}}, \quad J = \frac{\overline{C}}{\Delta C}\left(\frac{1}{\overline{C}} - I \right)$$

Here the terms representing averages and differences are

$$\overline{E}_l = \frac{E_{lb} + E_{la}}{2}, \quad \Delta E_l = \frac{E_{lb} - E_{la}}{2}$$

$$\overline{C} = \frac{C_{22} + C_{11}}{2}, \quad \Delta C = \frac{C_{22} - C_{11}}{2}$$

Such formula successfully eliminates the artificial strong anisotropy in several 2D semiconductors[1,4].

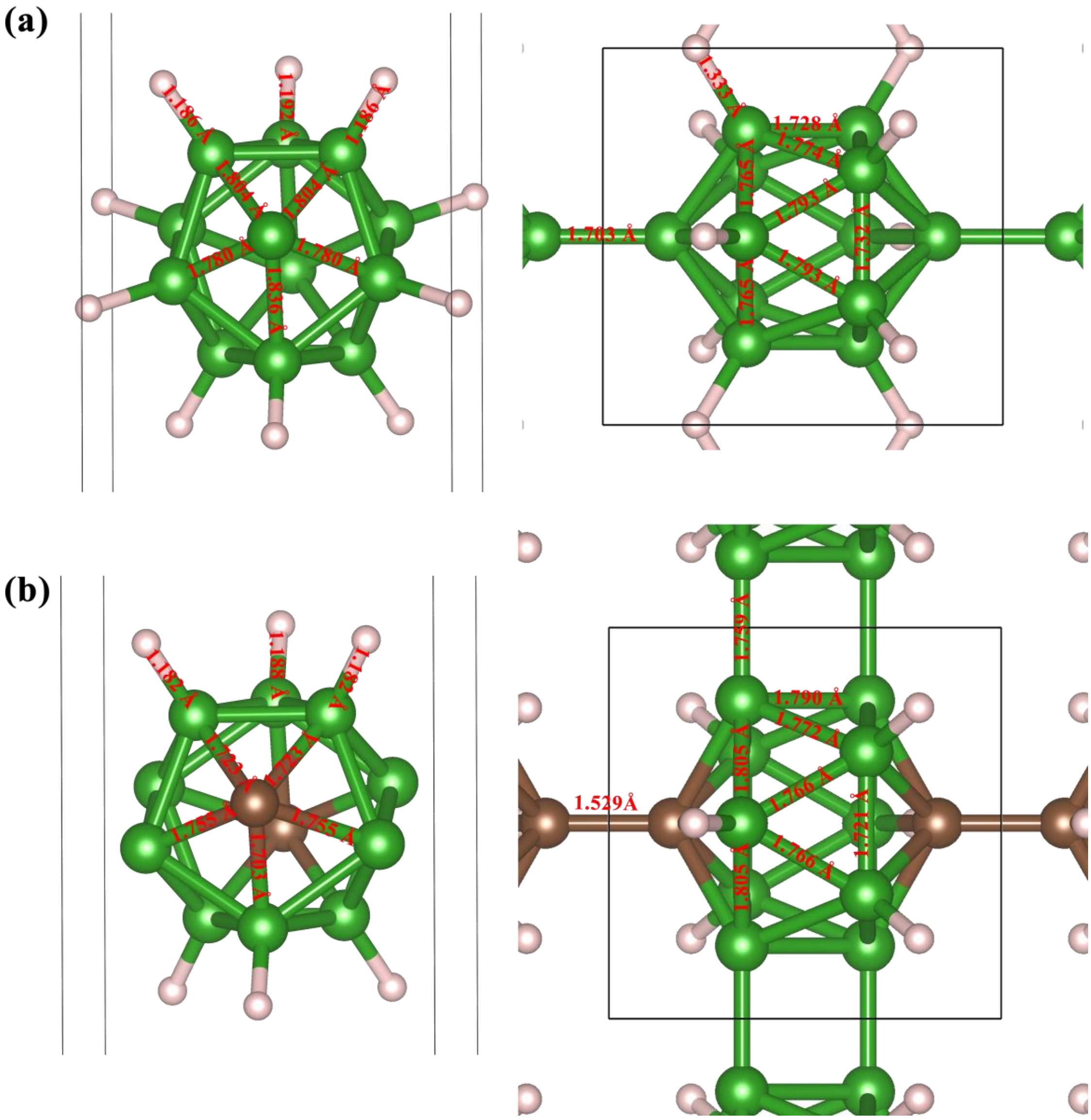

Figure S1. The bond length of (a) *o*-$B_{12}H_8$ and (b) *o*-$B_{10}C_2H_6$.

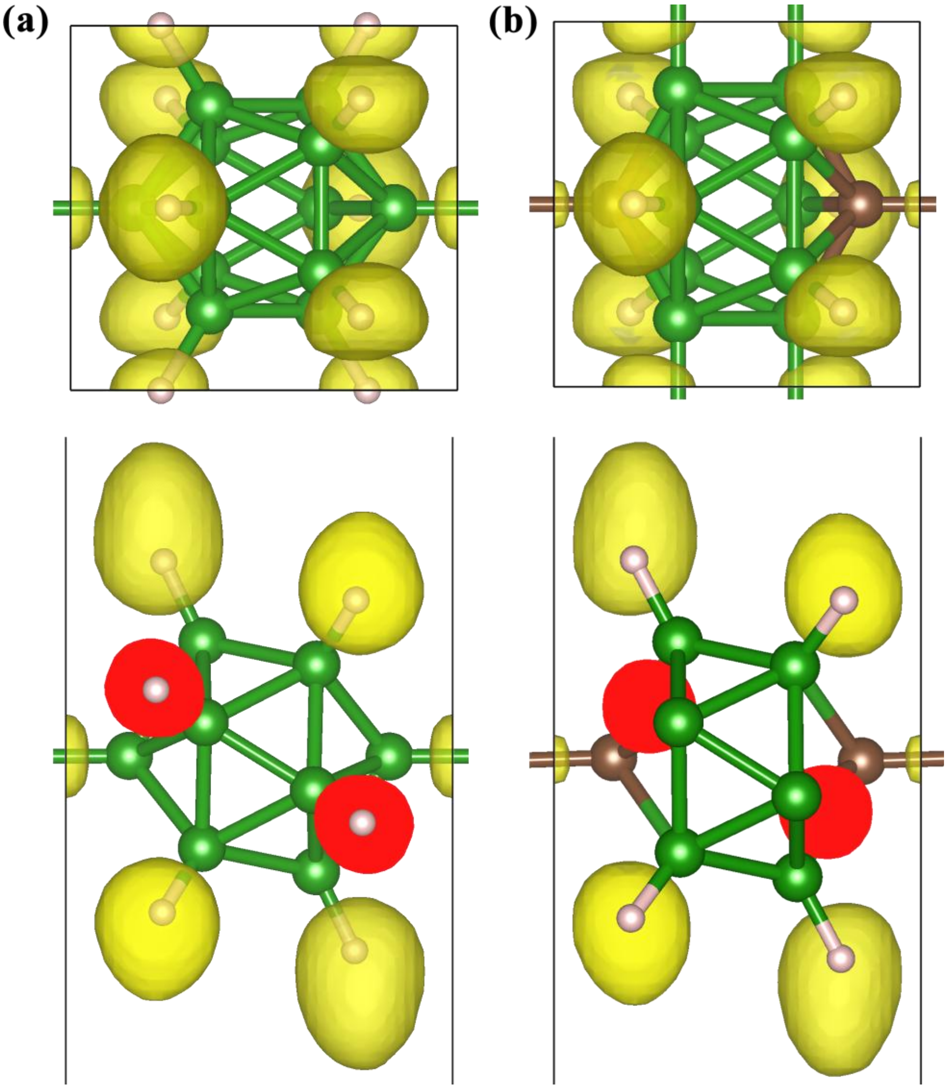

Figure S2. The electron localization function of (a) o-$B_{12}H_8$ and (b) o-$B_{10}C_2H_6$.

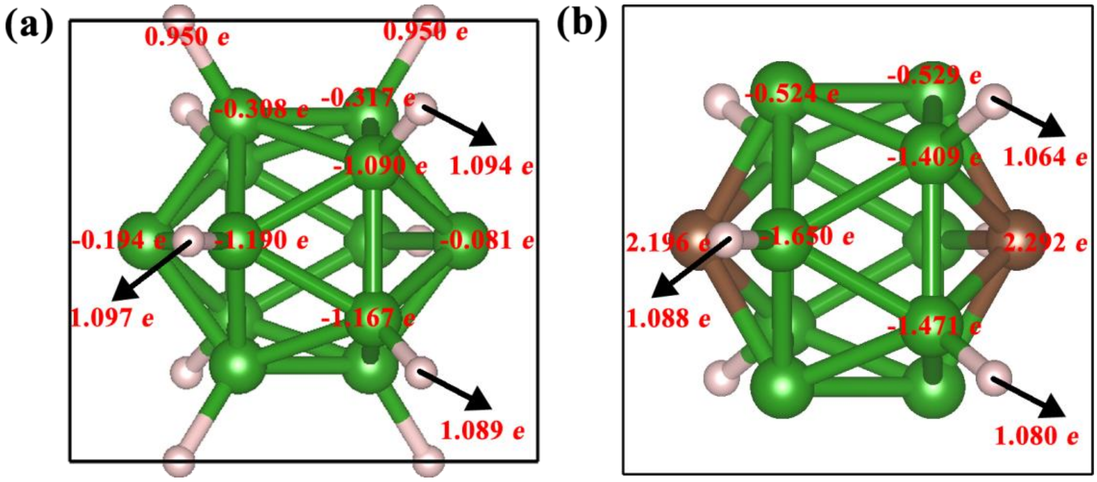

Figure S3. The Bader charge analysis results of (a) o-$B_{12}H_8$ and (b) o-$B_{10}C_2H_6$.

Table S1. Calculated axis Young's modulus (Y, N/m), axis Poisson's ratio (v), band gaps (GGA-PBE/HSE06, eV), carrier mobilities (m_x/m_y, $cm^2V^{-1}s^{-1}$) and Li/Na/K migration barriers (E_{ion}, eV) of o-$B_{12}H_8$ and o-$B_{10}C_2H_6$ MLs. The relevant parameters of the B_{12}-based 2D materials proposed in our previous work are also listed in the table for comparison.

Materials	Y_{11}/Y_{22}	Y_{max}/ Y_{min}	v_{11}/v_{22}	v_{max}/v_{min}	E_g^{PBE}/E_g^{HSE}	$m_x(e/h)$	$m_y(e/h)$	E_{ion}
o-$B_{12}H_8$	118.91/133.13	62.50	-0.009 /-0.01	0.498	3.56/4.75	7.487/249.583	2.806/322.265	0.084~0.615
o-$B_{10}C_2H_6$	194.13/233.67	109.18	0.008/0.01	0.490	3.58/4.63	2111.652/819.208	8440.619/8590.306	0.289~0.804
m-B_{24}[5]	179.64/149.35	183.77	0.152/0.126	0.054	0.528/1.296	0.83/1.77	1.71/0.93	0.16~0.89
o-B_{24}[5]	176.83/258.63	258.63	0.105/0.153	0.169	0.148/0.568	41.82/3.22	38.61/3.09	0.13~0.43
h-$B_{12}H_6$[6]	168.13	168.13	0.286	0.286	1.552/2.210	--	--	--
h-$B_{12}O_2H_6$[6]	207.45	207.45	0.234	0.234	3.746/4.927	810.54/44.40	269.75/1469.41	0.15~0.45
h-$B_{12}S_2H_6$[6]	130.31	130.31	0.213	0.213	4.196/5.257	258.41/57.49	635.46/47.29	0.033~0.23
h-$B_{12}N_2$[7]	161.77	161.77	0.273	0.273	0.057/0.995	58.40/1627.46	184.26/1560.22	--
h-$B_{12}P_2$[7]	95.80	95.80	0.305	0305	0.136/0.977	1113.68/2610.03	1072.66/2606.99	--
h-$B_{12}As_2$[7]	81.12	81.12	0.319	0.319	0.276/1.128	3410.55/24.34	1873.76/36.53	--

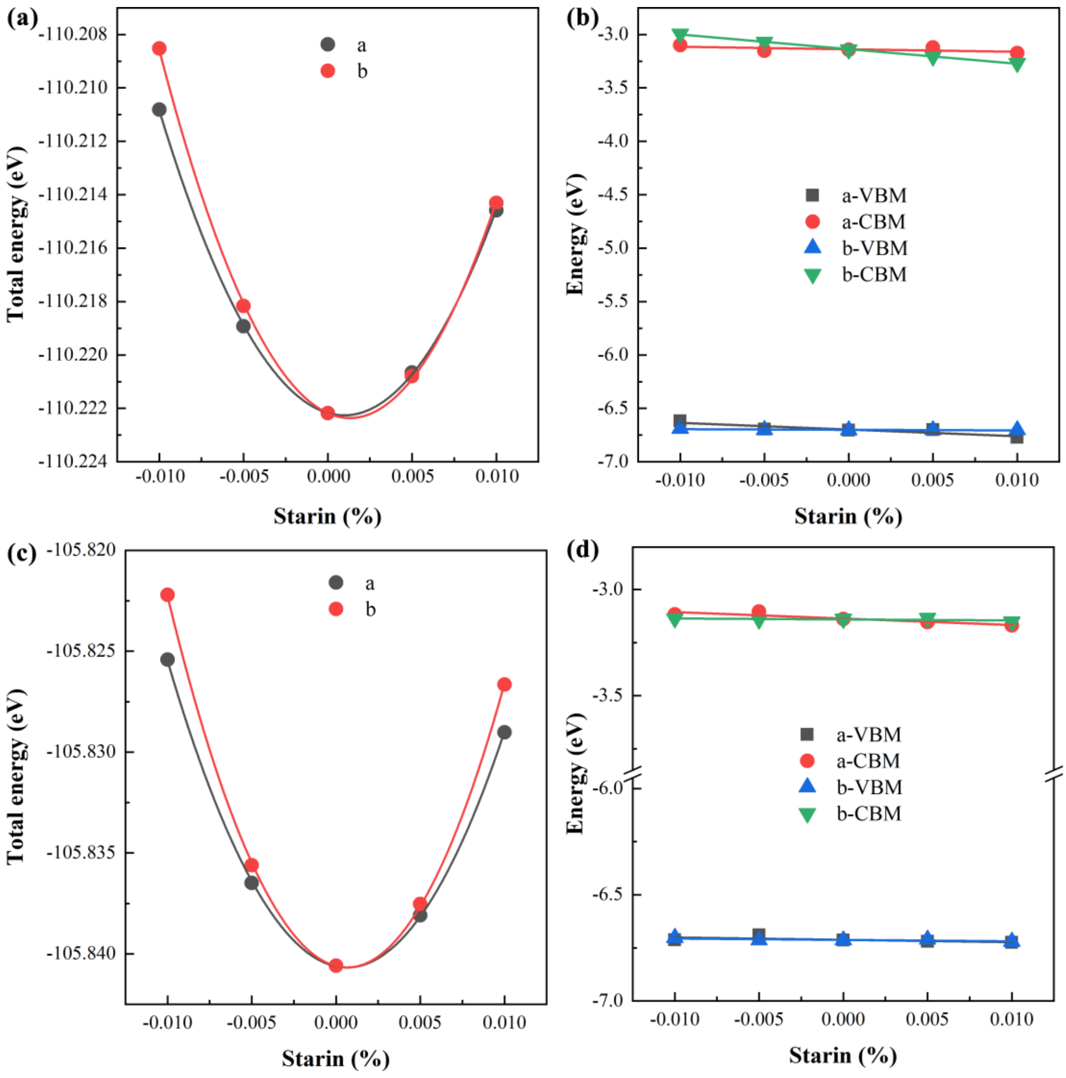

Figure S4. The fitting curves of the in-plane elastic modulus C_{2D} and the deformation potential constant E_1 of (a, b) *o*-$B_{12}H_8$ and (c, d) *o*-$B_{10}C_2H_6$ along *a* and *b* directions, respectively.

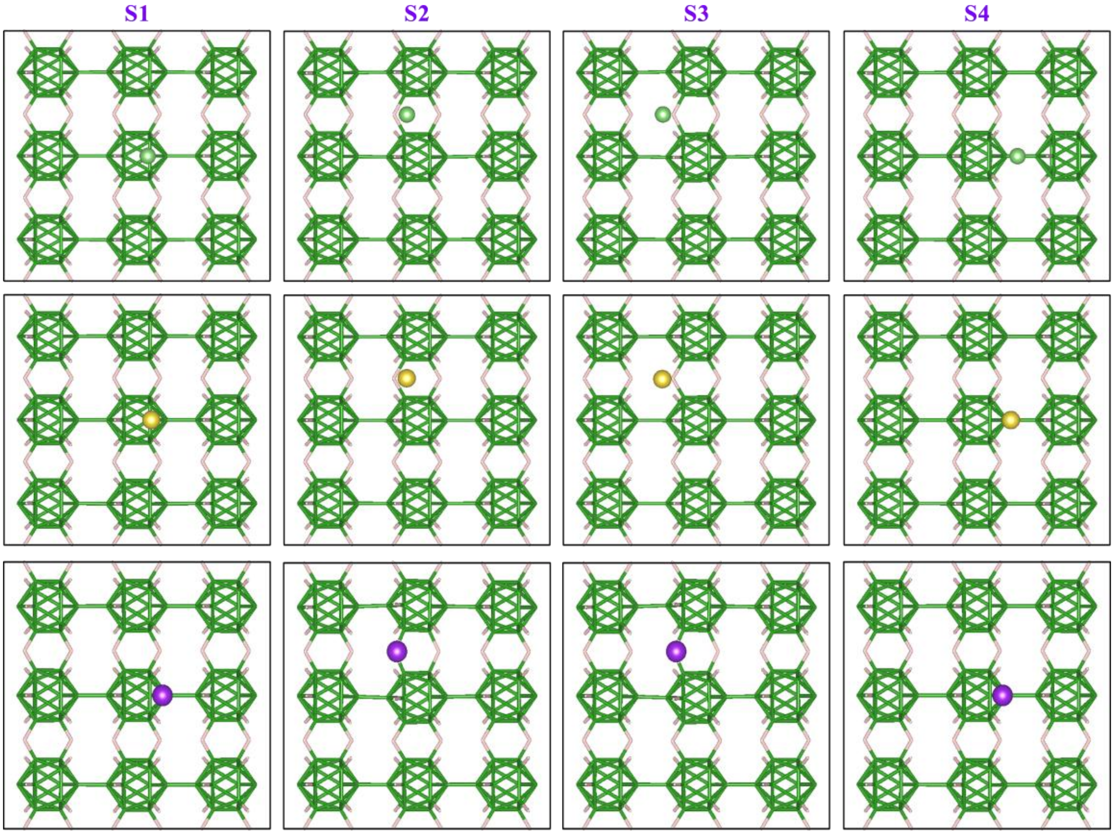

Figure S5. Optimized configurations of Li^+, Na^+, and K^+ at the various adsorption sites (S1–S4) on *o*-$B_{12}H_8$. Li^+ and Na^+ remain localized at their respective initial sites, whereas K^+ undergoes migration: the ions initially placed at S1 and S4 converge to the S4 configuration, while those at S2 and S3 converge to the equivalent stable sites above the bridging hydrogen atoms.

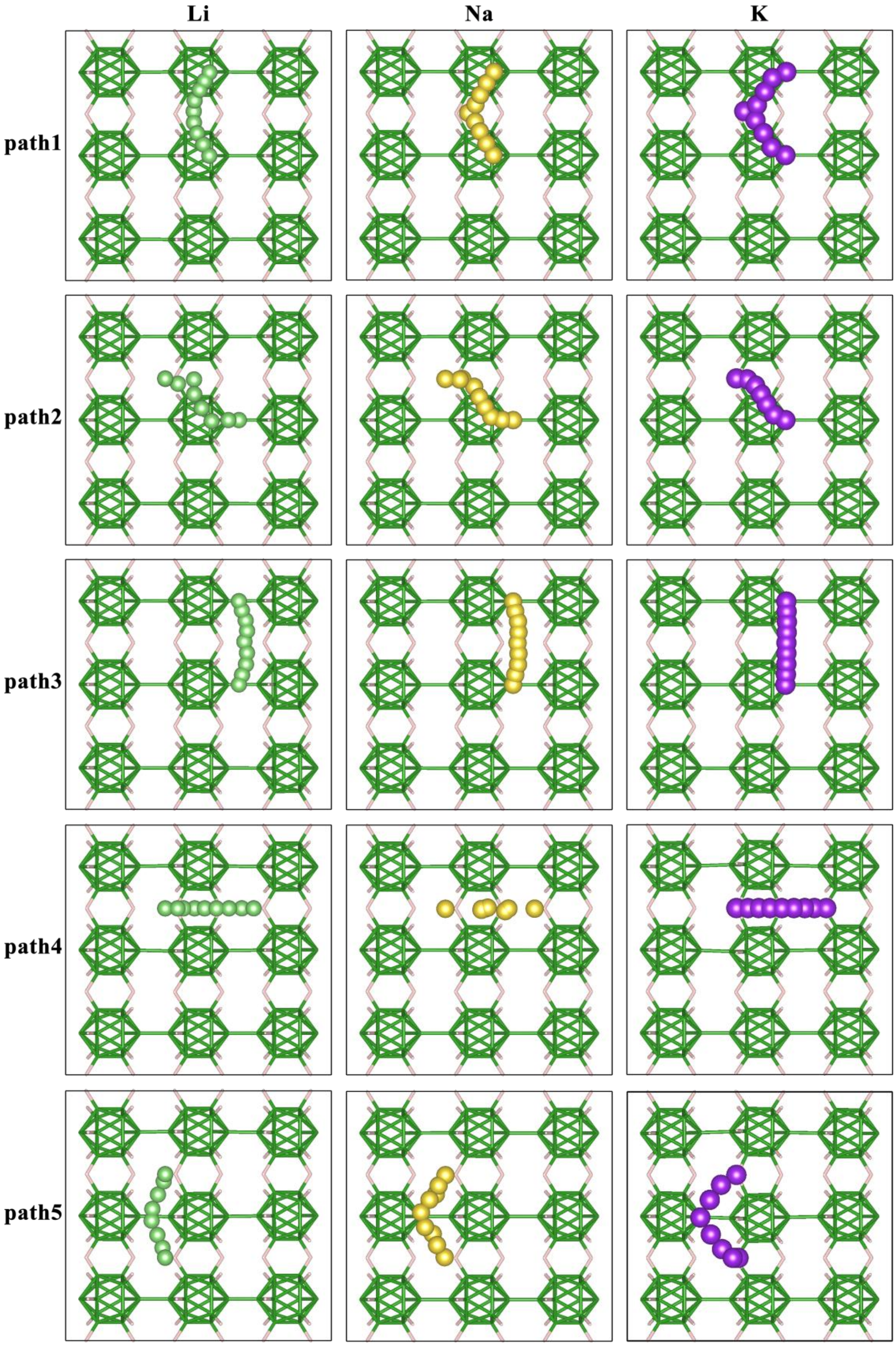

Figure S6. Ion migration diagrams of Li^+, Na^+, and K^+ along ~~four~~ five preset migration

paths on the o-$B_{12}H_8$ surface.

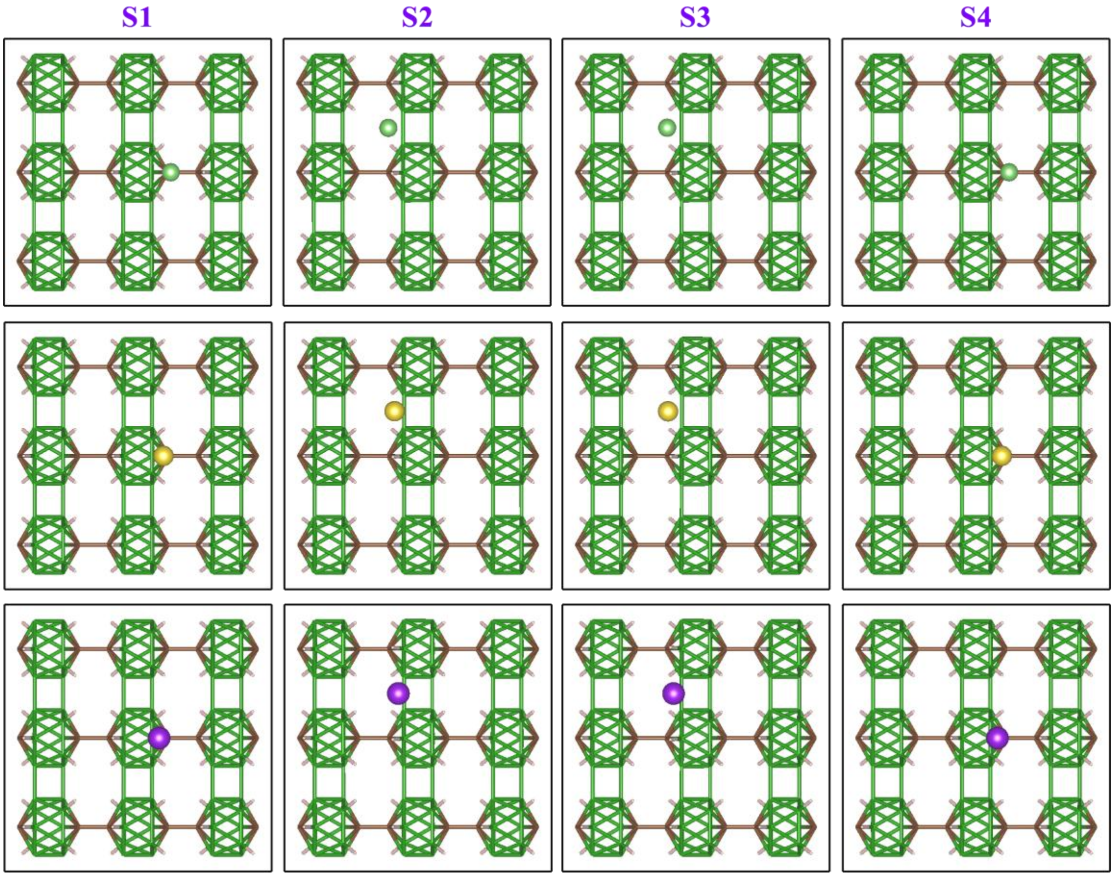

Figure S7. Optimized configurations of Li^+, Na^+, and K^+ at the various adsorption sites (S1–S4) on o-$B_{10}C_2H_6$. Li^+ and Na^+ remain localized at their respective initial sites, whereas K^+ undergoes migration: the ions initially placed at S1 and S4 converge to the S4 configuration, while those at S2 and S3 converge to the equivalent stable sites above the bridging hydrogen atoms.

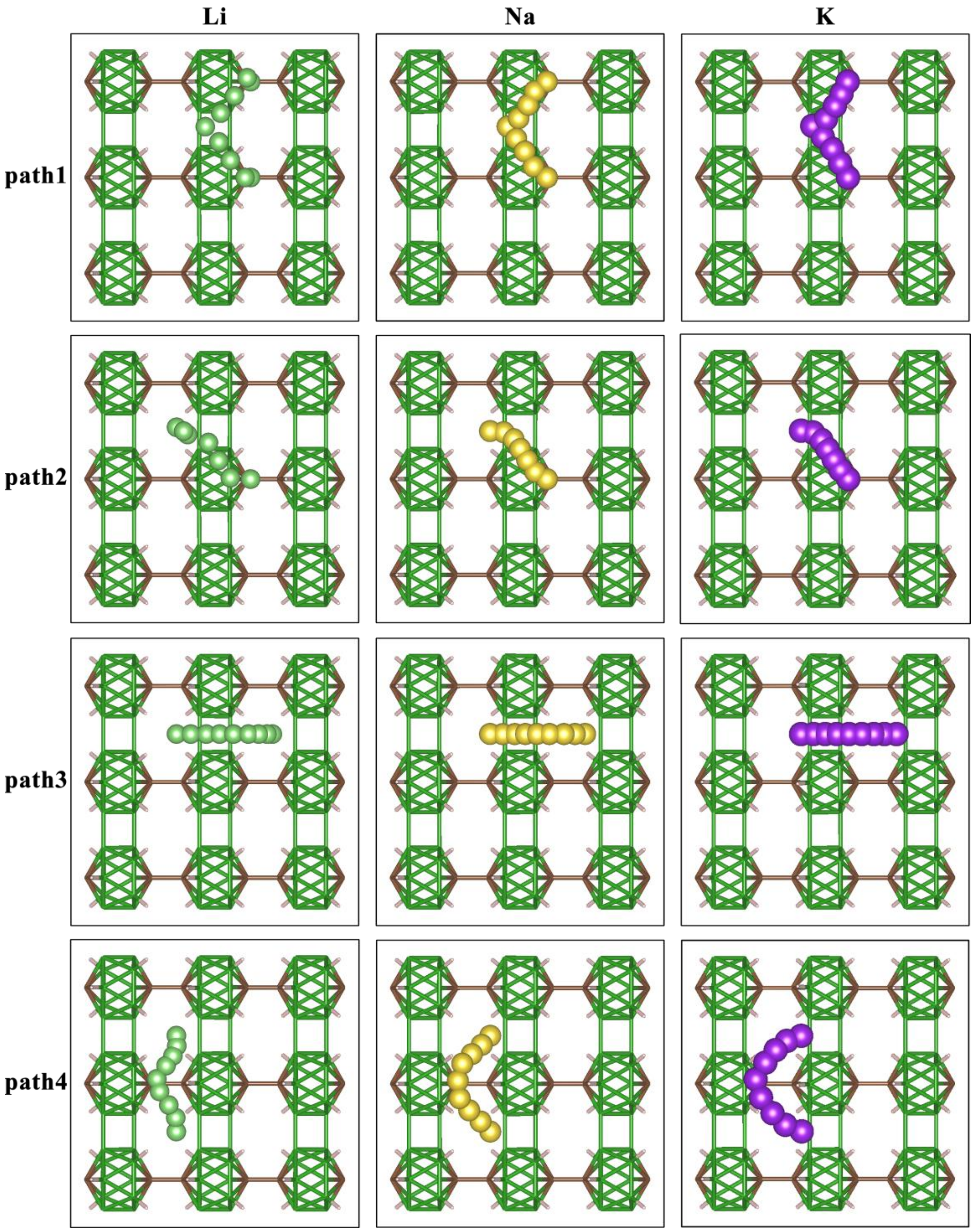

Figure S8. Ion migration diagrams of Li^{+}, Na^{+}, and K^{+} along four preset migration paths on the *o*-$B_{10}C_2H_6$ surface.

References

1. Yuan, J.-H.; Xue, K.-H.; Wang, J.-F.; Miao, X.-S. Gallium Thiophosphate: An Emerging Bidirectional Auxetic Two-Dimensional Crystal with Wide Direct Band Gap. *J. Phys. Chem. Lett.* **2019**, *10*, 4455–4462, doi:10.1021/acs.jpclett.9b01611.

2. Bardeen, J.; Shockley, W. Deformation Potentials and Mobilities in Non-Polar Crystals. *Phys. Rev.* **1950**, *80*, 72–80, doi:10.1103/PhysRev.80.72.
3. Lang, H.; Zhang, S.; Liu, Z. Mobility Anisotropy of Two-Dimensional Semiconductors. *Phys. Rev. B* **2016**, *94*, 235306, doi:10.1103/PhysRevB.94.235306.
4. Song, Y.-Q.; Yuan, J.-H.; Li, L.-H.; Xu, M.; Wang, J.-F.; Xue, K.-H.; Miao, X.-S. KTlO: A Metal Shrouded 2D Semiconductor with High Carrier Mobility and Tunable Magnetism. *Nanoscale* **2019**, *11*, 1131–1139, doi:10.1039/C8NR08046A.
5. Cao, Z.-G.; Wang, X.-Y.; Yuan, J.-H.; Wang, H.; Wu, G.-P.; Liu, Z.-H.; Wang, J. Designing Two-Dimensional Borophene from Icosahedral B12 Superatoms. *FlatChem* **2026**, *55*, 100985, doi:10.1016/j.flatc.2025.100985.
6. Gong, P.; Yuan, J.-H.; Wu, G.-P.; Liu, Z.-H.; Wang, H.; Wang, J. Designing 2D Wide Bandgap Semiconductor B12X2H6 (X=O, S) Based on Aromatic Icosahedral B12. *Nanomaterials* **2025**, *15*, 1803, doi:10.3390/nano15231803.
7. Gu, J.; Ding, M.-H.; Yuan, J.-H.; Wang, H.; Wu, G.-P.; Liu, Z.-H.; Wang, J. Designing a Novel Family of 2D B12X2 (X = N, P, As) Based on B12 Superatoms for Optoelectronic Application. *Materials Science in Semiconductor Processing* **2026**, *209*, 110653, doi:10.1016/j.mssp.2026.110653.
